# Gene Expression Profiling of Pulmonary Artery in a Rabbit Model of Pulmonary Thromboembolism

**DOI:** 10.1371/journal.pone.0164530

**Published:** 2016-10-31

**Authors:** Zhiyuan Tang, Xudong Wang, Jianfei Huang, Xiaoyu Zhou, Hao Xie, Qilin Zhu, Minjie Huang, Songshi Ni

**Affiliations:** 1 Department of Respiratory Medicine, Affiliated Hospital of Nantong University, Nantong, 226001, Jiangsu, China; 2 Department of Laboratory Medicine, Affiliated Hospital of Nantong University, Nantong, 226001, Jiangsu, China; 3 Department of Pathology, Affiliated Hospital of Nantong University. Nantong, 226001, Jiangsu, China; 4 Department of Clinical Bio-bank, Affiliated Hospital of Nantong University, Nantong, Jiangsu, China; 5 Key Lab of Drug Metabolism and Pharmacokinetics, State Key Laboratory of Natural Medicines, China Pharmaceutical University, Nanjing, Jiangsu, 210009, China; Cincinnati Children's Hospital Medical Center, UNITED STATES

## Abstract

Acute pulmonary thromboembolism (PTE) refers to the obstruction of thrombus in pulmonary artery or its branches. Recent studies have suggested that PTE-induced endothelium injury is the major physiological consequence of PTE. And it is reasonal to use PTE-induced endothelium injury to stratify disease severity. According to the massive morphologic and histologic findings, rabbit models could be applied to closely mimic the human PE. Genomewide gene expression profiling has not been attempted in PTE. In this study, we determined the accuracy of rabbit autologous thrombus PTE model for human PTE disease, then we applied gene expression array to identify gene expression changes in pulmonary arteries under PTE to identify potential molecular biomarkers and signaling pathways for PTE. We detected 1343 genes were upregulated and 923 genes were downregulated in PTE rabbits. The expression of several genes (IL-8, TNF-α, and CXCL5) with functional importance were further confirmed in transcript and protein levels. The most significantly differentially regulated genes were related to inflammation, immune disease, pulmonary disease, and cardiovascular diseases. Totally 87 genes were up-regulated in the inflammatory genes. We conclude that gene expression profiling in rabbit PTE model could extend the understanding of PTE pathogenesis at the molecular level. Our study provides the fundamental framework for future clinical research on human PTE, including identification of potential biomarkers for prognosis or therapeutic targets for PTE.

## Introduction

Acute pulmonary thromboembolism (PTE) is the most common form of pulmonary embolism (PE), which refers to the obstruction of thrombus in the pulmonary artery or its branches. Worldwide, PTE is a major contributor to global noncommunicable disease burden with considerably high mortality and morbidity[1,2]. Traditionally, PTE is more prevalent in developed countries than in developing countries, with its incidence increasing along with the aging of the population[3]. Despite the lower annual incidence of PTE in Asia populations[4,5], PTE has been increasing recently due to the elevated life expectancy in these countries. Recent studies in Asian countries have indicated that PTE rate among hospitalized patients is approaching the rates observed in Western countries[6].

The main pathology of PTE is pulmonary artery hypertension, hypoxia and hemodynamic instability. When the right ventricular load significantly rises, right side cardiac failure may develop with hypotension[7,8]. PTE is also a common cause of pulmonary vascular endothelium injury. Vascular endothelium cells (VECs) act as the mechanical barrier between the circulating blood and the smooth muscle in the vascular wall, with normal ones being critical for maintaining vascular permeability and regulated inflammatory reaction.

During PTE manifestation, thrombi trapped in pulmonary vessels would damage the vascular endothelium, thus causing unregulated release of proinflammatory mediators[7,9]. In addition, endothelial progenitor cells are mobilized from bone marrow to the circulation to repair damaged endothelium. It has been shown that pulmonary vascular remodeling triggered by repeated vascular injuries of the pulmonary vessels may lead to secondary pulmonary hypertension[10], which is the main clinical consequence of PTE. Therefore, it has been hypothesized that PTE-induced endothelium injury plays a crucial role in the pathophysiological consequences of PTE[1]. However, a study in children does not show evidence of persistent pulmonary hypertension after PE[11].

Several studies have investigated the expression changes of plasma biomarkers in pulmonary artery during PTE. Brain natriuretic peptide (BNP) as well as N-terminal pro-BNP (NT-proBNP) in blood has been identified as biomarkers to predict echocardiographic right ventricular (RV) dysfunction in patients with acute PTE[12,13]. Troponin I and D-dimer have also been reported to growin PTE patients[14]. In addition, Celik et al identified increased level of plasma Tenascin-C among acute PTE patients[15]. However, genome-wide gene expression profiling of pulmonary artery tissues of PTE patients has not been analyzed yet.

In the current study, we first determined whether the rabbit autologous thrombus model accurately represents human PTE disease. Then we analyzed gene expression changes of pulmonary artery during acute rabbit model. The goal of the study is to find out endothelial gene expression changes in PTE and perhaps further to identify candidate biomarkers that may play important roles in the disease. At the same time, the study is engaged in the assessment of PTE disease severity, paving the foundation for future PTE clinical research.

## Materials and Methods

### Ethics Statement

All animal experiments were approved by the Animal Ethics Committee of Affiliated Hospital of Nantong University (Nantong, China). This research was performed in strict accordance with the guidelines for animal experimentation of this institution. We made every effort to minimize animal suffering, distress and pain. When the rabbit become inaction, the fur loses luster and looks dull, we consider the rabbits were through distressing. And we had in place a protocol for early/humane endpoints. When rabbits became breathless, with low heart beat or low blood pressure, the animals were sacrificed. We tried to reduce the number of animals being used in our study. The procedures of animal experiments were carried out as humanely as possible.

### Animals and PTE model establishment

Animals were maintained in standard raised-flooring cages at approximately 25°C and 12-h light and 12-h dark in the Department of Animal Center, Medical College of Nantong University. Twenty healthy China big-ear rabbits, specific-pathogen-free (SPF) grade and weighing between 2.5 and 3 kg, were purchased from the Center of experimental animals of the Nantong University. These rabbits were randomly divided into control (n = 10) and experimental group (n = 10). One hour before the experiment, thrombi were generated by collecting 0.5 ml blood from the marginal ear vein and placing into a sterile petri dish for 45 minutes at room temperature. The autologous blood clot was subsequently cut into 5 mm sized aliquots and suspended in sterile saline. We disinfected the operation site with iodine and alcohol before surgical procedures to minimize infection, we used sodium pentobarbital (3ml/kg, IV) through marginal ear vein injection for anesthesia during surgical procedures to alleviate suffering. After anesthesia, a venous catheter was placed in the exposed and isolated femoral vein. In the experimental group, the blood clot in 10 mL of saline was injected into the femoral vein. An additional 5 mL of saline were used to flush the catheter. In the control group, 15 mL of saline were injected into the femoral vein. After operation, we first made sure there was no bleeding, then sutured the skin, disinfected the site with iodine., The rabbits were left in the case until they recover. The rabbits were monitored three times every day. Before animal sacrificed, 1 ml blood was collected through ear vein, serum was prepared through centrifugation, and stored at -80°C. After blood draw, heparin was given at 1000U/kg through ear vein.

### Tissue and blood sample collection

Seven days after the injection of autologous blood clot, the animals were given heparin 1 mL/kg via the marginal ear vein before being sacrificed with an overdose of mobarbital (2 mL/kg). Blood was drawn from the pulmonary artery, centrifuged at 3,000/min at 4°C for 10 minutes. The serum was stored in -80°C. The pulmonary arteries were ligated just before entry into the lungs, dissected out and rinsed with saline. The pulmonary artery from one side was fixated in 10% neutral formalin or 48 hours and the artery from the other side was stored in -80°C. Bilateral pneumonectomy was performed, the lungs were inspected for infarct. The infarcted tissue was removed and fixated in formalin in 48 hours.

### Morphological Analysis

Tissue sections (4 um) of the formalin-fixated pulmonary artery and infarcted lung tissue were prepared following routine procedures of water rinse, sequential dehydration, paraffin embedding, sectioning on microtome and H&E staining. The H&E slides were baked in 65°C oven for 2 hours before microscopic examination.

Eight random microscopic fields of the lung parenchyma were selected for morphologic analysis of the pulmonary arteries by the HPIAS 1000 high-definition system. The extrapulmonary artery was examined for luminal narrowing and endothelial denudation.

### RNA isolation and gene expression profiling

RNA was isolated from frozen pulmonary artery using the Trizol method. Total RNA from each sample was quantified using the NanoDrop ND-1000 and the RNA integrity was assessed using standard denaturing agarose gel electrophoresis. For microarray analysis, Agilent Array platform was employed. The sample preparation and microarray hybridization were performed according to the manufacturer’s standard protocols. Briefly, total RNA from each sample was amplified and transcribed into fluorescent cRNA with using the manufacturer’s Agilent’s Quick Amp Labeling protocol (version 5.7, Agilent Technologies). The labeled cRNAs were hybridized onto the Whole Rabbit Genome Oligo Microarray covering 14,765 genes (4x44K, Agilent Technologies). Finally, the hybridized arrays were scanned by the Agilent Scanner G2505C. A total of 8 RNA samples (4 control samples and 4 experimental samples) were analyzed by the expression microarray.

Agilent Feature Extraction software (version 11.0.1.1) was used to analyze acquired array images. Agilent probe IDs were mapped to Ensembl Gene IDs by bioDBnet[16]. Quantile normalization and subsequent data processing were performed using the GeneSpring GX v11.5 software package (Agilent Technologies). After quantile normalization of the raw data, genes that at least 4 out of 8 samples have flags in Detected (“All Targets Value”) were chosen for further data analysis. Differentially expressed genes with statistical significance were identified through Volcano Plot filtering. Finally, gene clustering analysis was performed using J-express and MeV with Pearson distance and average linkage[17,18].

### Functional analyses of differentially expressed genes

In order to analyze functional relevance of differentially expressed genes, we first converted rabbit gene IDs (Oryctolagus cuniculus, OryCun 2.0) to human homologous gene IDs (Homo sapiens, GRCh38.p5) using BioMart based on Ensembl release 83 (http://www.biomart.org/)[19]. For one-to-many mapping genes, we selected the gene with the highest sequence homology.

Functional enrichment analysis of differentially expressed genes (DEGs) were performed using the WebGestalt online tool[20]. To identify statistically significant enriched terms among GO, KEGG and disease databases, the total genes interrogated were set as the background and an adjusted P value of 0.05.

We have reported our microarray dataset in the following repository: Gene Expression Omnibus, No. GSE84738 (NCBI tracking system #17972531)

### Expression validation by qRT-PCR and western blot analysis

IL-8, TNF-α, CXCL5 and β-actin mRNA levels were determined by quantitative reverse transcription PCR (qRT-PCR)[21]. The primers used were as follows: IL-8 forward primer (5’- ATG ACT TCC AAG CTG GCC GTG GCT-3’) and reverse primer (5’-TCT CAG CCC TCT TCA AAA ACT TCT C -3’); TNF-α forward primer (5’- CGA GTG ACA AGC CTG TAG C -3’) and reverse primer (5’-CCT TCT CCA GCT GGA GAG C-3’); CXCL5 forward primer (5’-GAG AGC TGC GTT GCG TTT G-3’) and reverse primer (5’-TTT CCT TGT TTC CAC CGT CCA-3’); GAPDH forward primer (5’-ACCACAGTCCATGCCATCAC-3’) and reverse primer (5’-TCCACCACCCTGTTGCTGTA-3’). Sample input was normalized to β-actin mRNA expression. IL-8, TNF-α, CXCL5 and β-actin protein levels were determined by western blot analysis[22], using following antibodies: ab34100 (1:1000 dilution, abcam, Cambridge, MA) for IL-8, ab199013 (1:1000 dilution, abcam, Cambridge, MA) for TNF-α, ENA-78 (1:1000 dilution, Santa Cruz Biotechnology, Santa Cruz, CA) for CXCL5 and ab8226 (1:500 dilution, abcam, Cambridge, MA) for β-actin.

### Statistical analysis

All data were expressed as the mean ±standard deviation (SD). The statistical significance of differences between groups was determined using a two-tailed Student’s t-test and one-way ANOVA. A probability (p) less than 0.05 is considered statistically significant. Each experiment consisted of at least three replicates per condition.

## Results

### Rabbit model recapitulates pathogenesis of human PTE

**Fig 1:**
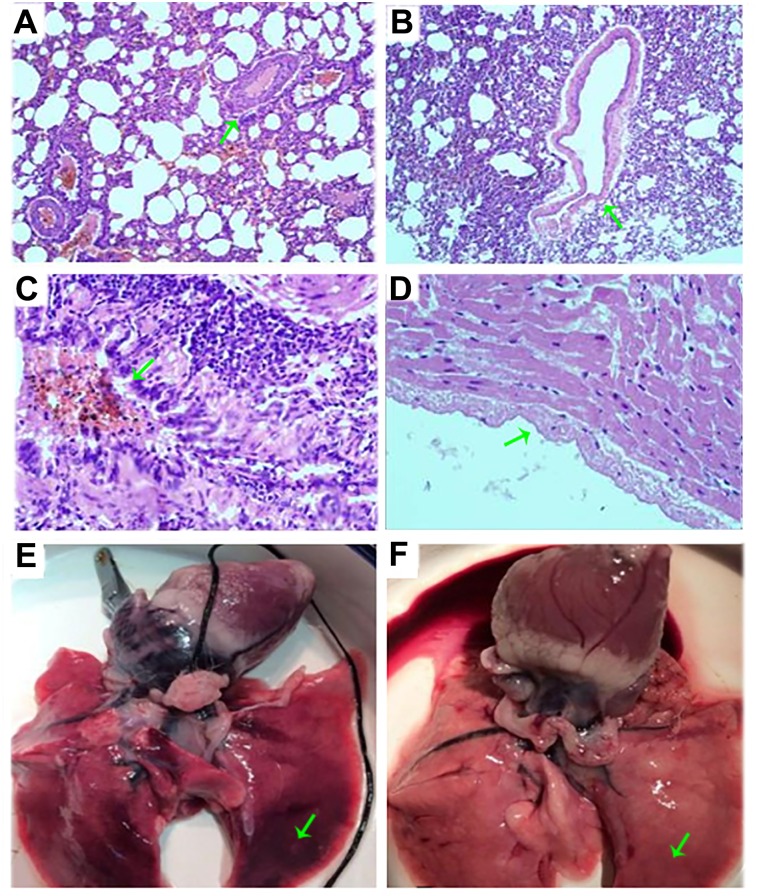
H&E staining of pulmonary arteries and photos of the lung from control and PTE rabbits. (A) and (C): microscopic H&E staining of pulmonary arteries from PTE rabbit, 100 x and 400 x amplification respectively; (B) and (D): microscopic H&E staining of pulmonary arteries from control rabbit, 100 x and 400 x amplification respectively; E: gross appearance of lung tissue from PTE rabbit; F: gross appearance of lung tissue from control rabbit.

At the time of sacrifice, PTE rabbits had lower body weight (2200g±400g vs 2500g±500g), diminished physical activity, yellow hair and reduced appetite when comparing to the control group rabbits. Of gross appearance, the lungs from PTE rabbits were unevenly inflated. Adhesion between lung base and pleura could be detected in some cases as well as wedge shaped infarct in the apex pointing to the hilum. The embolus could be seen in the cross section of pulmonary artery (Fig 1E and 1F). Microscopically, embolus was found in pulmonary arterioles. The endothelial hyperplasia and neutrophilic infiltrates were observed in the pulmonary artery in the PTE rabbits (Fig 1A–1D). Two rabbits died prior to the experimental endpoint, one due to anesthesia overdose, the other due to pulmonary embolism. In summary, both gross and microscopic observations of lung and pulmonary artery from PTE rabbits resembled the pathogenesis of human PTE.

### PTE leads to dramatic gene expression changes in pulmonary artery tissues

**Fig 2:**
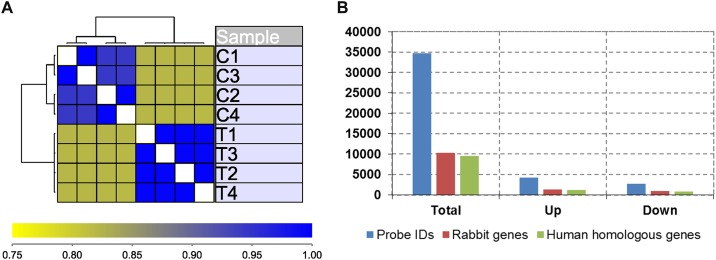
Gene expression profiling pulmonary arteries from control and PTE rabbits. (A) Unsupervised clustering of gene expression datasets of pulmonary artery from four control and four PTE rabbits. (B) Total number of probes, rabbit gene numbers and matched homologous human genes on the expression array, as well as numbers of upregulated and downregulated genes in PTE rabbits compared to control rabbits.

Hierarchical clustering analysis grouped datasets based on their experimental groups (Fig 2A), and box plot within each experimental group (S1 Fig) indicated good reproducibility within the group and significant difference between groups. To identify differentially expressed genes in PTE pulmonary arteries, we performed a volcano plot filtering between the two groups with different cutoff values (FDR<0.05, FC>2) (S2 Fig). Of 10, 356 Ensembl genes interrogated on the array in which a total of 1343 genes were up-regulated and 923 genes were down-regulated (Fig 2B, S1 Data).

### Functional analysis of differentially expressed genes associated with PTE

**Fig 3:**
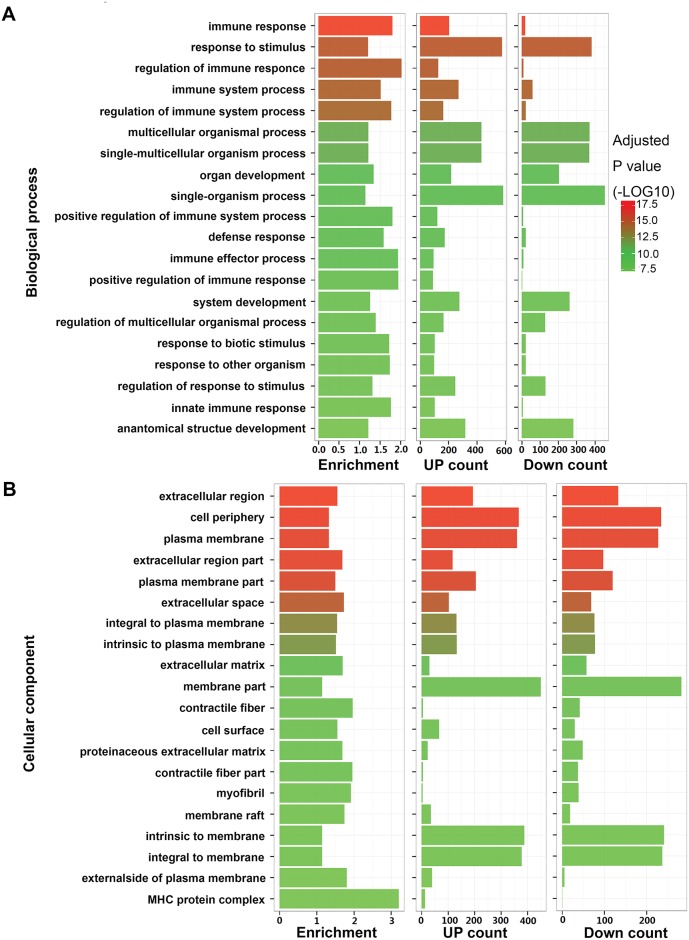
Functional analysis of differentially expressed genes in PTE pulmonary artery. (A) Top twenty enriched biological processes. (B) Top twenty enriched cellular components. From left to right: enrichment fold, number of upregulated genes, and number of downregulated genes. Different P values are indicated by different colors.

Of overall differentially expressed genes, the top twenty biological processes and cellular components were listed in Fig 3. Four out of the top five biological processes were involved in immune system (immune response, regulation of immune response, immune system process, and regulation of immune system process) And four out of the top six cellular components were associated with extracellular components (extracellular region, cell periphery, extracellular region part, and extracellular space). In addition, of these significantly enriched functions and pathways, there were more upregulated genes than downregulated genes (S2 Data). And content of the plasma brain natriuretic peptide (BNP) and N-terminal pro-BNP (NT-proBNP) of the control and PTE rabbits were determined(S1 Table). Plasma levels of BNP and NT-proBNP of PTE rabbits increased significantly than control rabbits (p <0.05).

The enriched KEGG signaling pathways in up- or down-regulated genes included immune disease signaling pathway, cell adhesion molecules (CAMs) pathways (S3 Fig), and cytokine-cytokine receptor interaction (S4 Fig) [23–38]. Of upregulated genes, the top KEGG signaling pathways included T cell receptor signaling pathway, NOD-like receptor signaling pathway, RIG-I-like receptor signaling pathway, Chemokine signaling pathway (S6 Fig), Fc epsilon RI signaling pathway, Toll-like receptor signaling pathway (S5 Fig)[39–50], and B cell receptor signaling pathway (Table 1). The top KEGG signaling pathway of downregulated genes was Calcium signaling pathway (Table 1).

**Table 1:** Enriched signaling pathways in upregulated and downregulated genes in PTE pulmonary artery.

Regulation	KEGG ID	Description	Gene count	P value
**Up-regulated**	4660	T cell receptor signaling pathway	23	4.0E-04
**Up-regulated**	4621	NOD-like receptor signaling pathway	13	1.6E-03
**Up-regulated**	4622	RIG-I-like receptor signaling pathway	13	3.0E-03
**Up-regulated**	4062	Chemokine signaling pathway	25	4.0E-03
**Up-regulated**	4664	Fc epsilon RI signaling pathway	14	6.8E-03
**Up-regulated**	4620	Toll-like receptor signaling pathway	14	2.2E-02
**Up-regulated**	4662	B cell receptor signaling pathway	13	2.6E-02
**Down-regulated**	4020	Calcium signaling pathway	19	2.1E-02

**Fig 4:**
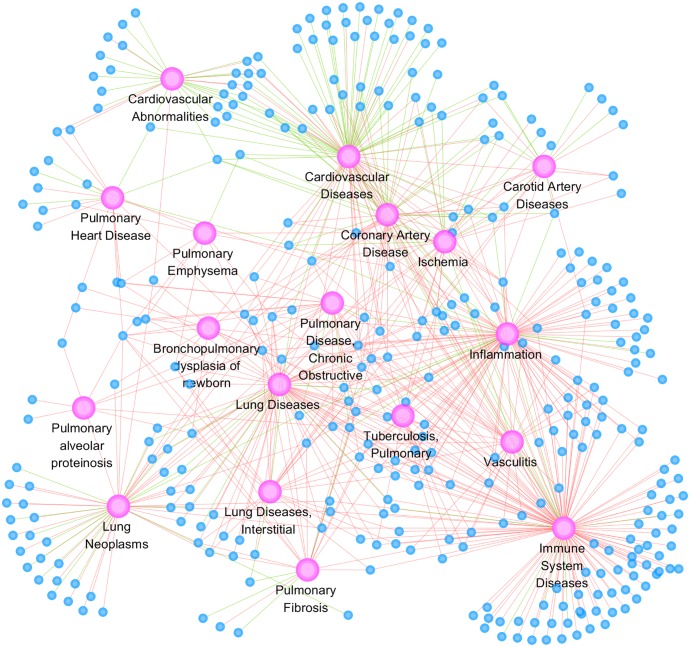
Disease network of differentially expressed genes in PTE pulmonary artery. Eighteen lung and cardiovascular disease categories are used to construct the network. Purple circles indicate disease categories, blue circles indicate differentially expressed genes. Red lines indicate upregulation and green lines indicate downregulation.

To further elucidate the underlying disease mechanism, we also determined the relationship between differentially expressed genes in PTE and cardiovascular and lung disease categories. Eighteen disease categories were used in the disease network construction (Fig 4), including 11 lung disease categories, 5 cardiovascular disease categories, and 2 immune disease categories.

### Validation of differentially expressed genes by quantitative reverse transcription PCR (qRT-PCR), western blot analysis and immunohistochemistry analysis

**Fig 5:**
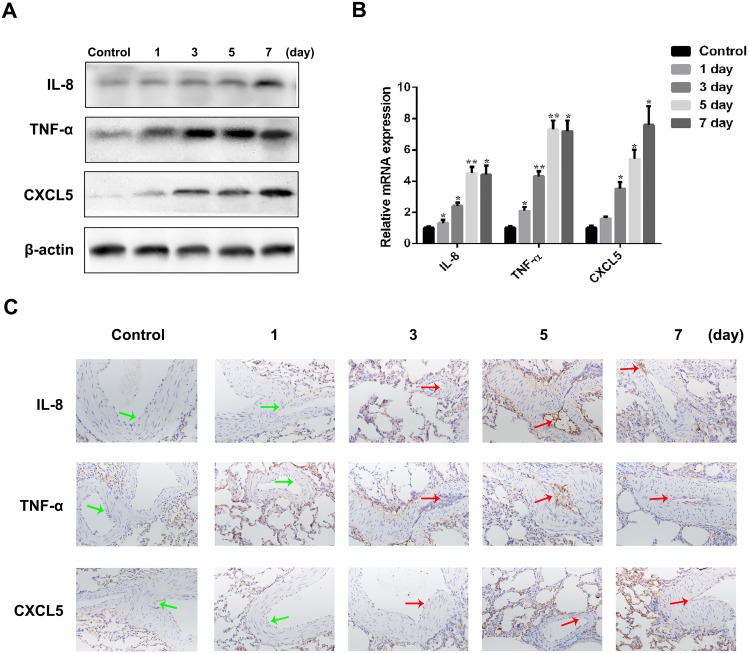
Validation of selected upregulated genes in PTE pulmonary arteries by western blot analysis, qRT-PCR, and immunohistochemistry analysis. (A)Western blot analysis of IL-8, TNF-α, and CXCL5 protein expression during PTE progression. (B) qRT-PCR analysis of IL-8, TNF-α and CXCL5 mRNA expression during PTE progression. Actin was used as an internal control. All of the data are shown as the mean±S.E.M.; * p<0.05, ** p<0.01 compared with the corresponding control group; n = 3. (C) IL-8, TNF-α, and CXCL5 proteins were detected by immunohistochemistry in endothelial cells.

To validate differentially expressed genes, we determined both mRNA and protein expression of three genes (IL-8, TNF-α, and CXCL5) by qRT-PCR and western blot analysis respectively. The expression of all three genes significantly increased by time after the induction of PTE when compared to control normal rabbits (Fig 5A and 5B), which was further confirmed by qRT-PCR, western blot analysis and immunohistochemistry analysis in endothelial cells. (Fig 5C)

## Discussion

In the current study, we performed genome-wide gene expression profiling of pulmonary arteries in a rabbit PTE model. We detected dramatic changes in gene expression in PTE rabbit and three genes (IL-8, TNF-α, and CXCL5) were subsequently confirmed by qRT-PCR and western blot analysis. The most significantly regulated genes in a differential manner were related to cardiomyopathy and inflammation. Our results are consistent with recent studies reporting significant changes of pulmonary artery during PTE[51–53].

The importance has been underscored in recent studies of the crosstalk between pulmonary endothelial cells and thrombi in the pathogenesis of PTE. In both PTE animal models and PTE patients, alternations of pulmonary endothelium function have been observed, such as procoagulant activity and fibrinolytic function, along with a noticeable increase in the blood concentration of plasminogen activator inhibitor type I and arginase II protein in PTE patients[51,54]. On one hand, PTE might lead to the dysfunction of endothelial cells through both mechanical and inflammatory insults, with both the lysing thrombus and mechanical effects of thrombotic occlusion damaging the endothelium of the vein[55]; while on the other hand, considering the necessity ofendothelial cells in the resolution of venous thrombi, dysfunctional endothelial cells may contribute to the further progression of the PTE diseases[56,57]. Our data confirmed previous hypothesis that PTE induced dramatic gene expression alterations in pulmonary artery. However, two alternative hypotheses above cannot be distinguished through the data, emphasizing the future mechanistic studies to determine whether inflammatory changes caused by dysfunction of endothelial cells are thrombogenic.

Our gene expression profiling data suggests that more genes are upregulated than downregulated during PTE: at cutoff of 2 fold (FDR<0.05), 1343 genes were upregulated and 923 genes were downregulated in PTE rabbits (Fig 2B). Of eight enriched signaling pathways, seven pathways were upregulated (Table 1). The data is consistent with a recent bioinformatic study on gene expression analysis of lung tissues during PTE[52], suggesting that upregulation of gene expression is more prevalent during PTE. The result has presented significant implications in PTE diagnosis and treatment. Similar to the lung tissue gene expression analysis during PTE, it is also identified the enrichment of T cell receptor signaling pathway, cytokine-cytokine receptor interaction signaling pathway, NOD-like receptor signaling pathway, chemokine signaling pathway and Toll-like receptor signaling pathway.

The data is consistent with existing literatures on the relationship between inflammation and PTE. It was reported more than 30 years ago that white blood cell count was significantly associated with incidence and mortality of coronary heart disease[58]. Recently, both epidemiological and clinical studies have shown that inflammatory and immune response play important roles in pulmonary embolism (PE): gene expression of type I and II IFN was significantly associated with PE[59]; protein levels of C-reactive protein (CRP), fibrinogene, and leukocyte count were associated with chronic obstructive pulmonary disease (COPD)[60]; gene expression profiling of lung tissues in PE has identified differentially expressed genes enriched in inflammatory, defense and immune response pathways[53].

During the selection of the top candidates, the mRNA expression of 65 genes was first detected that had greatly changed in the three pathways in rabbits between the control and PTE rabbits through RT-PCR, and 10 with the greatest changes were selected among them. Secondly, the protein expression changes of these 10 genes were also spotted by the Westernblot method, and three molecules were finally selected, which were IL-8, TNF-α and CXCL5, in combination with the experimental and literature retrieval results. Cytokines and chemokines are important cell signaling mediators of acute inflammation, adaptive cellular and humoral immune responses[61]. Increased expression of both CXC and C-C chemokines were detected in pulmonary thromboembolism lung tissues[62,63]. In the current study, we validated the upregulation of three chemokines (IL-8, TNF-α and CXCL5) by qRT-PCR and western blot analysis in PTE arteries. IL-8 is a potent neutrophil attractant and activator mainly secreted by macrophages and plays a role in innate immune response. Circulating IL-8 level is higher in patients with idiopathic venous thrombosis[64]; IL-8 is involved in acute lung injury and acute respiratory distress syndrome through the generation of IL-8 and IL-8 autoantibody complexes and influence neutrophil apoptosis[65]; IL-8 level was significantly higher in patients in the acute myocardial infarction stages of coronary artery disease[66]. TNF-α is mainly produced by activated macrophages, which is an important marker for systemic inflammation. Blood level of TNF-α also remained significantly higher in patients with PE[67] and chronic obstructive pulmonary disease (COPD). CXCL5 is another key chemoattractant and activator of neutrophils., who mediates the circadian neutrophil recruitment to the lung[68], with higher expression in patients with coronary artery disease[69].

Limits are surely posed in the study. In our rabbit PTE model, although autologous blood clot formation is similar to thrombosis, autologous blood clots do not laminate permanently like real thrombi[55], thus the imitation of human disease might not be fully reached. Therefore, our findings should be validated in human patients. Second, we only analyzed gene expression changes after 7 days, a time course experiment should be included in the future to distinguish primary from secondary gene expression changes.

## Conclusions

PTE remains a significant cause of death in hospitalized patients. Better understanding of the molecular and cellular pathophysiology may allow improved therapies to lessen the early mortality and long-term morbidity of PTE[53]. Our results suggest that PTE does cause pulmonary artery gene expression changes, which might be used for prognosis or potential therapeutic targets for PTE.

## Supporting Information

S1 Fig Box plot of gene expression profile datasets of control and PTE rabbit pulmonary arteries. (TIF)

S2 Fig Volcano plot of gene expression profiling in control and PTE pulmonary arteries. (TIF)

S3 Fig Cell adhesion molecules (CAMs) signaling pathway. Genes upregulated in PTE pulmonary artery are indicated in blue color and genes downregulated are indicated in purple color.
(TIF)

S4 Fig Cytokine-cytokine receptor interaction signaling pathway. Genes upregulated in PTE pulmonary artery are indicated in blue color and genes downregulated are indicated in purple color.
(TIF)

S5 Fig Toll-like receptor signaling pathway. Genes upregulated in PTE pulmonary artery are indicated in blue color and genes downregulated are indicated in purple color.
(TIF)

S6 Fig Chemokine signaling pathway. Genes upregulated in PTE pulmonary artery are indicated in blue color and genes downregulated are indicated in purple color.
(JPG)

S1 Data Total gene analysis data. (XLS)

S2 Data Gene function analysis data. (XLS)

S1 Table Content of the plasma brain natriuretic peptide (BNP) and N-terminal pro-BNP (NT-proBNP) of the model. (DOCX)
