# Supplementary figures and images for: Gene Expression Profiling of Pulmonary Artery in a Rabbit Model of Pulmonary Thromboembolism
Source: PLoS One. 2016 Oct 31;11(10):e0164530. doi: 10.1371/journal.pone.0164530 (PMC5087918; doi:10.1371/journal.pone.0164530)

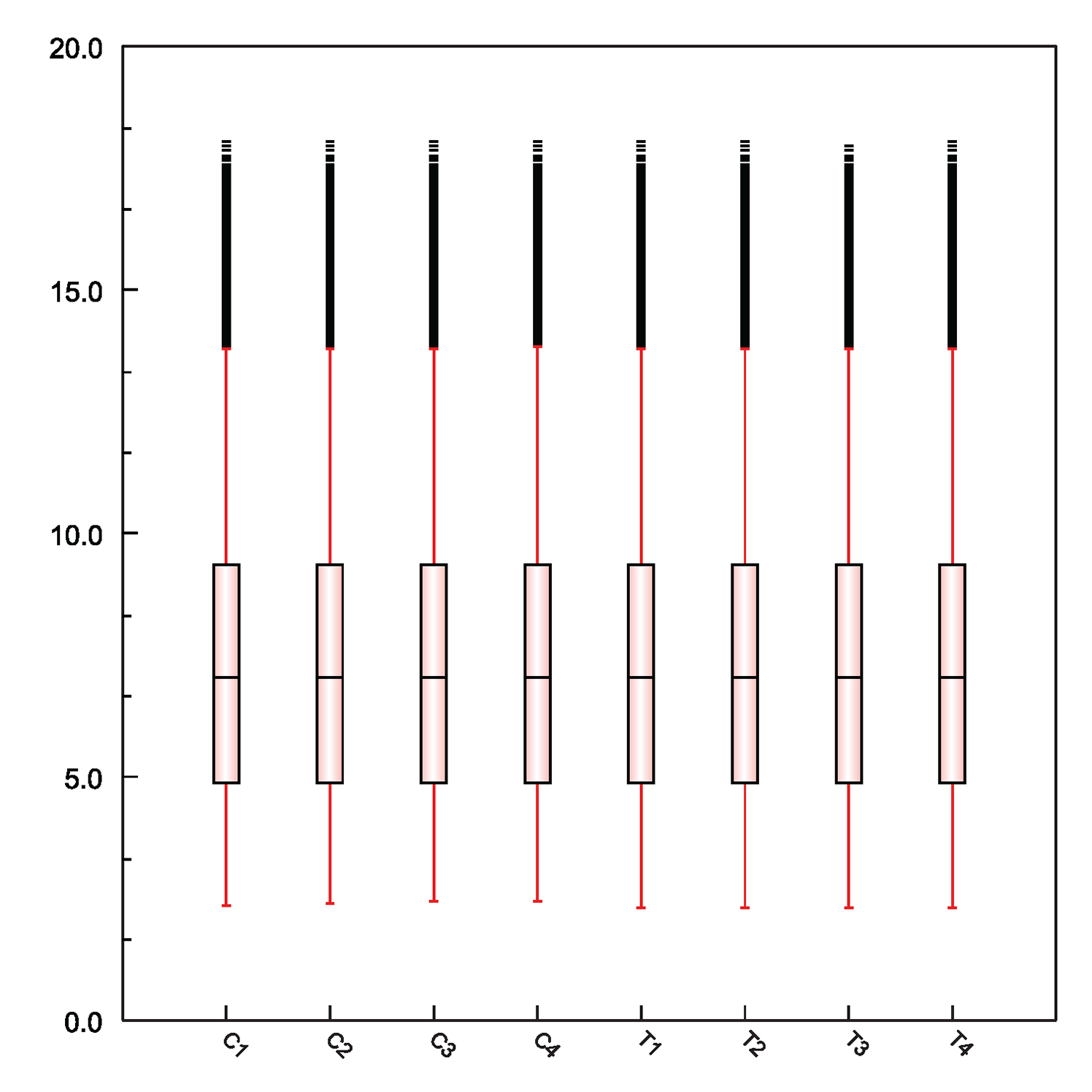

Supplement: S1 Fig — (TIF) [file pone.0164530.s001.tif]

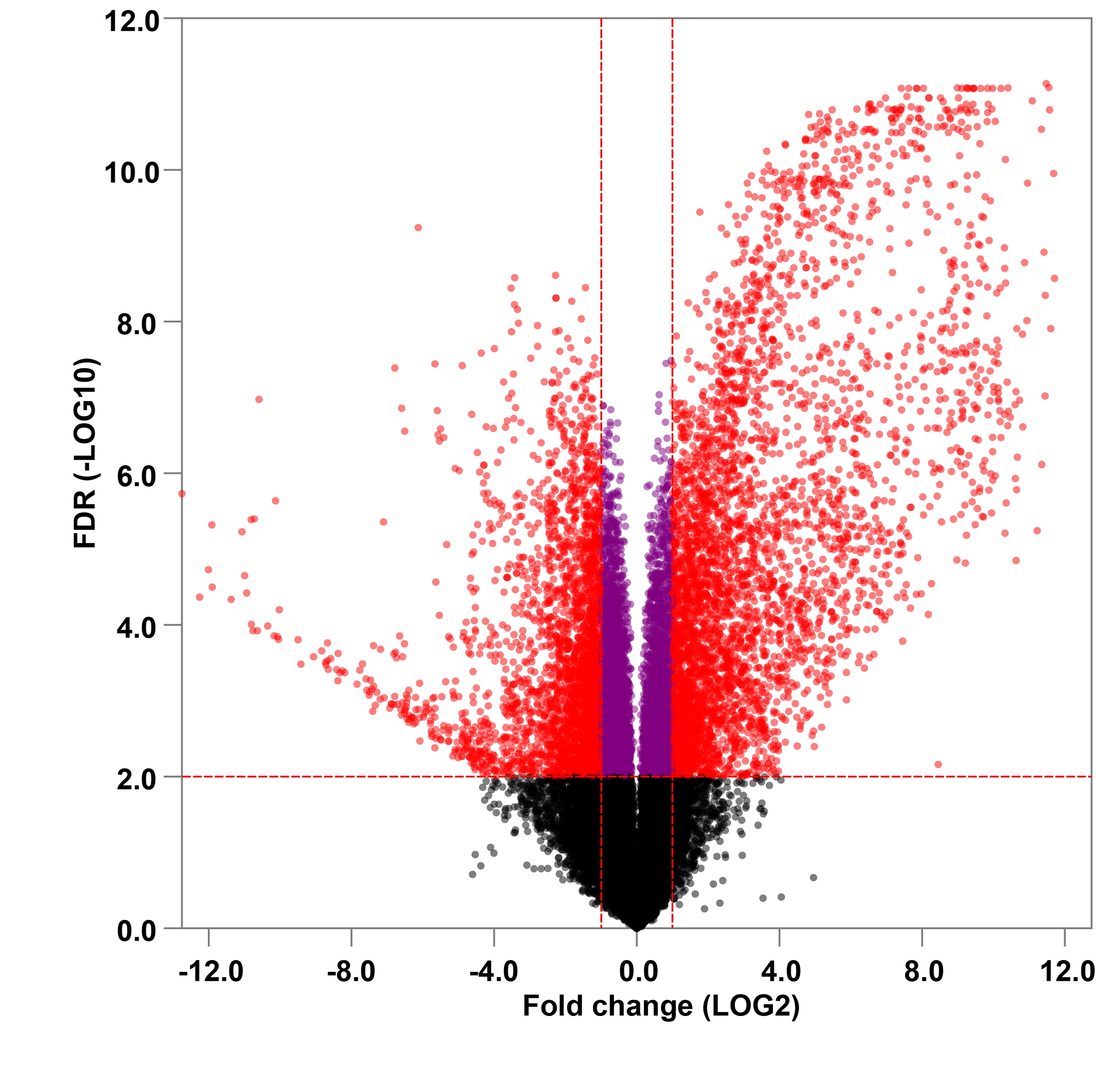

Supplement: S2 Fig — (TIF) [file pone.0164530.s002.tif]

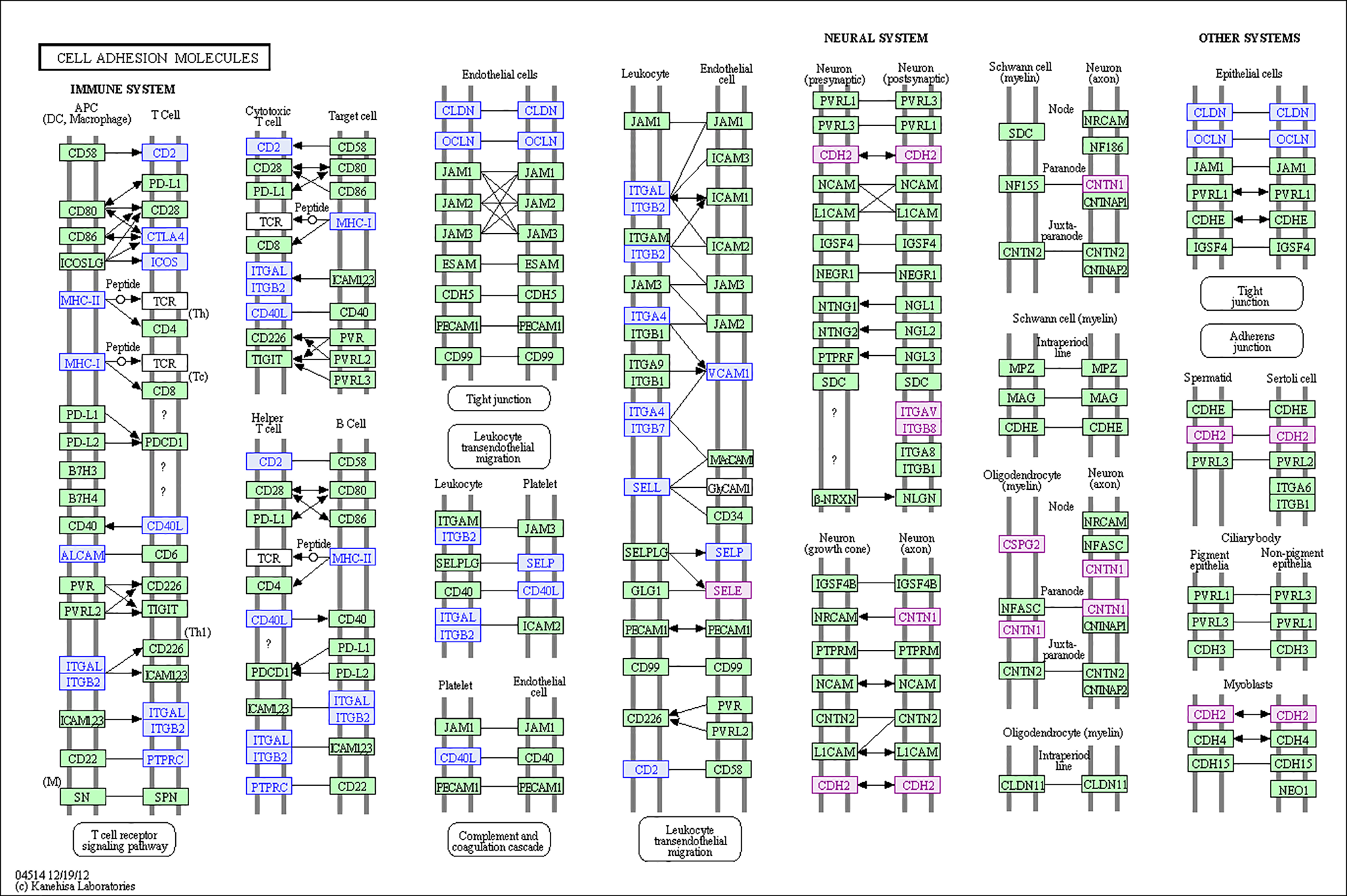

Supplement: S3 Fig — Genes upregulated in PTE pulmonary artery are indicated in blue color and genes downregulated are indicated in purple color. (TIF) [file pone.0164530.s003.tif]

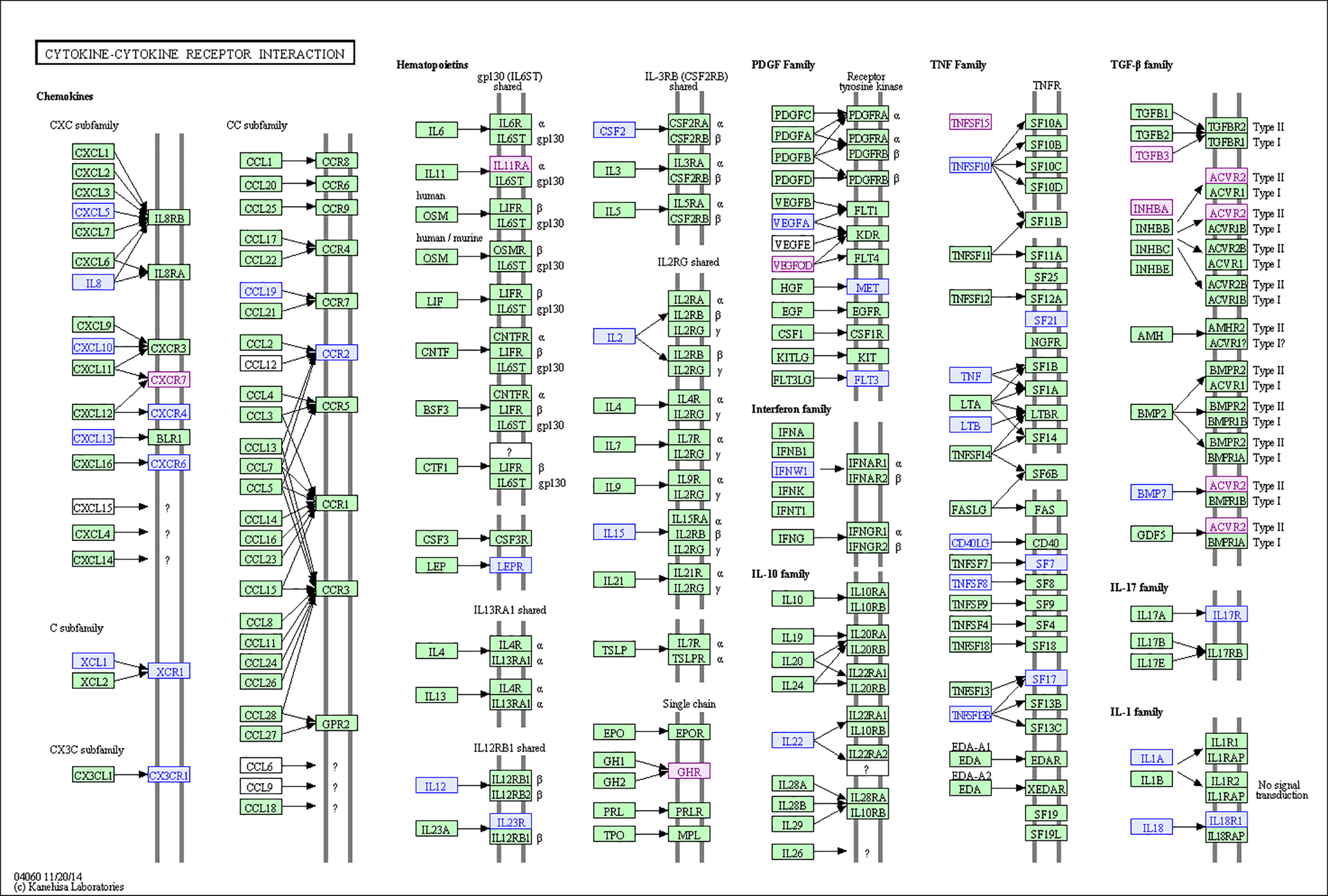

Supplement: S4 Fig — Genes upregulated in PTE pulmonary artery are indicated in blue color and genes downregulated are indicated in purple color. (TIF) [file pone.0164530.s004.tif]

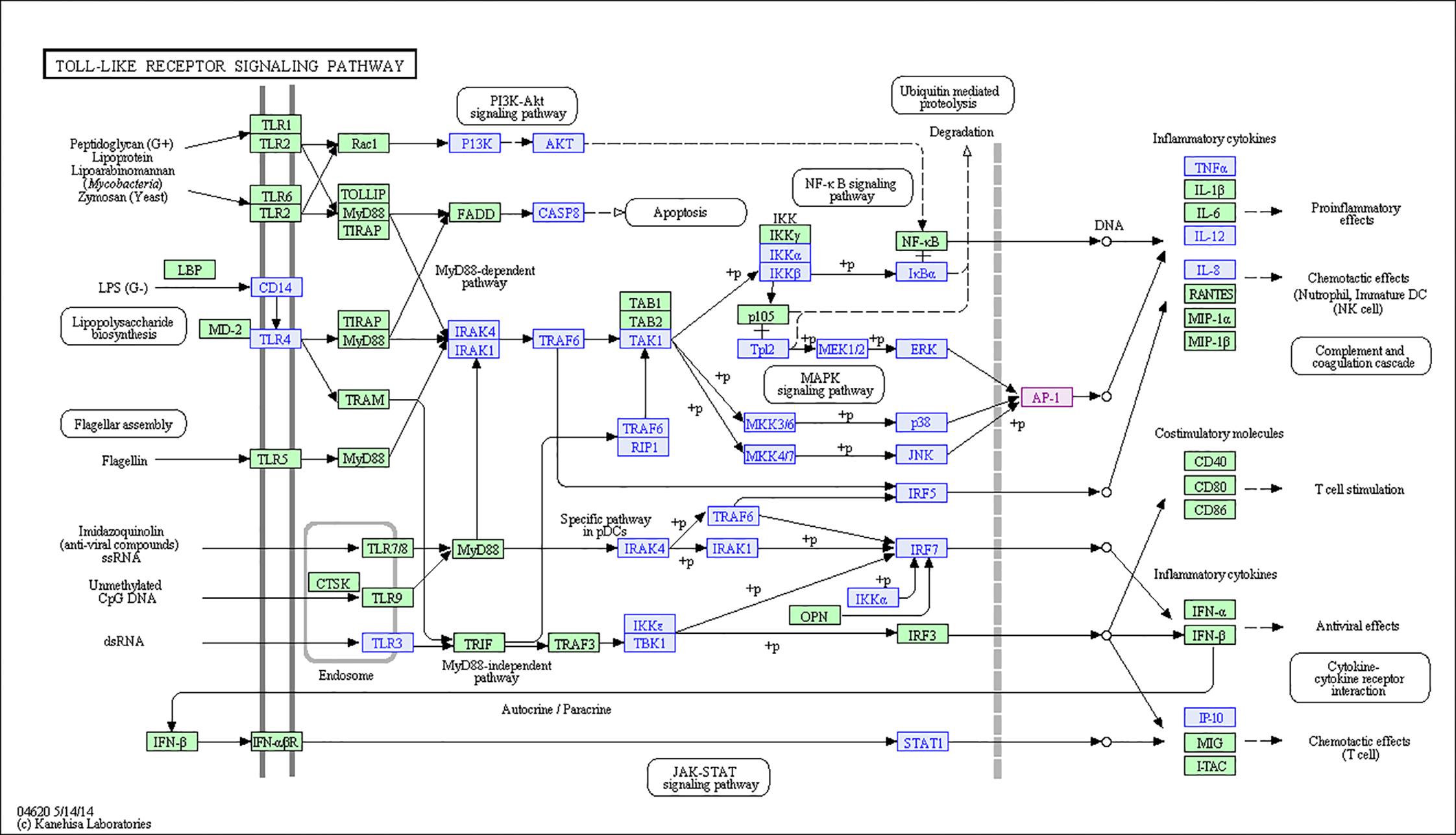

Supplement: S5 Fig — Genes upregulated in PTE pulmonary artery are indicated in blue color and genes downregulated are indicated in purple color. (TIF) [file pone.0164530.s005.tif]

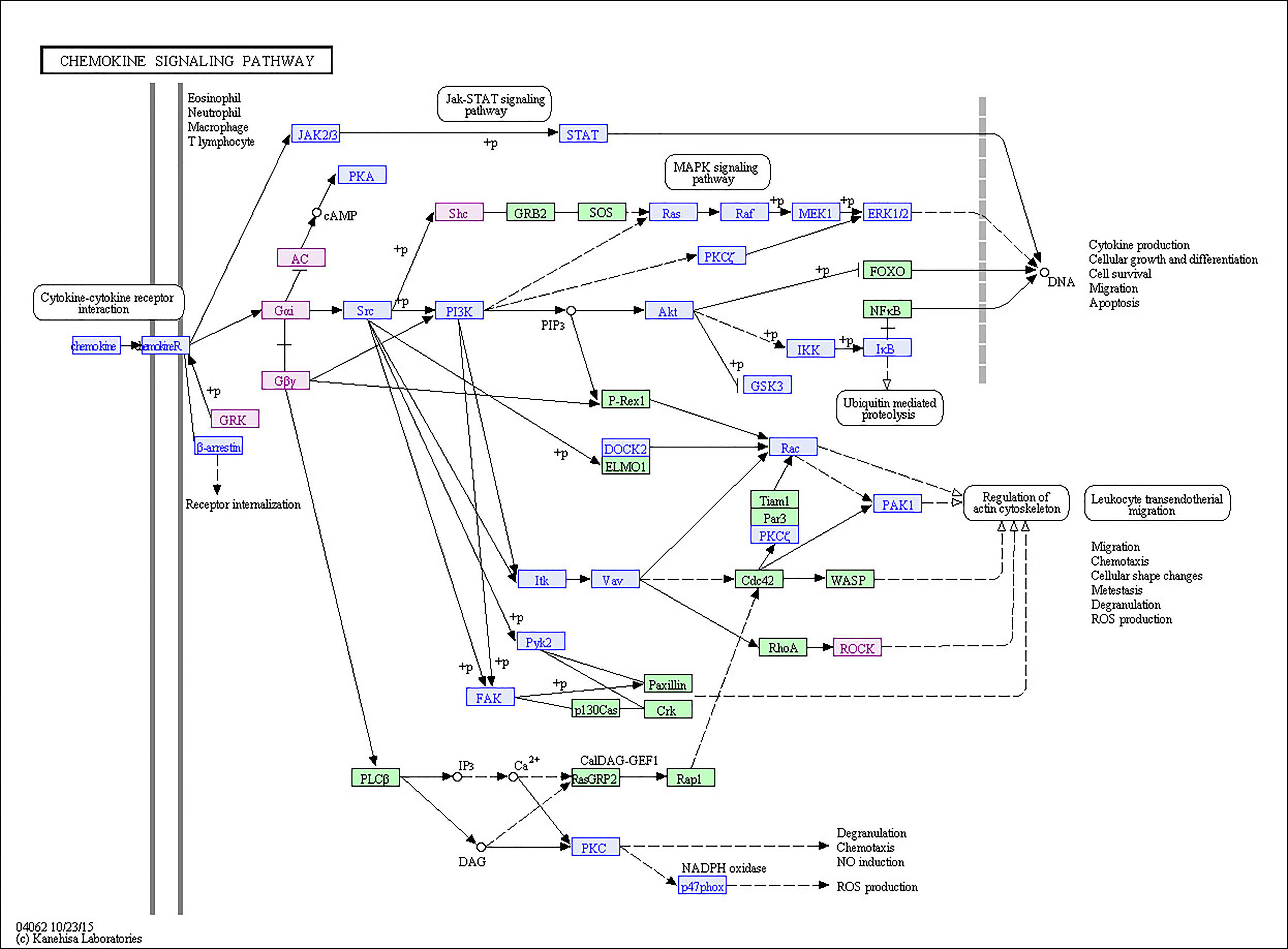

Supplement: S6 Fig — Genes upregulated in PTE pulmonary artery are indicated in blue color and genes downregulated are indicated in purple color. (JPG) [file pone.0164530.s006.jpg]
