# Supplementary material for: Gene Expression Profiling of Pulmonary Artery in a Rabbit Model of Pulmonary Thromboembolism
Source: PLoS One. 2016 Oct 31;11(10):e0164530. doi: 10.1371/journal.pone.0164530 (PMC5087918; doi:10.1371/journal.pone.0164530)
Supplement: S1 Table — (DOCX) [file pone.0164530.s009.docx]

Supplementary Table 1. Content of the plasma brain natriuretic peptide (BNP) and N-terminal pro-BNP (NT-proBNP) of the model

| Group | Heart Rate  (times/min) | BNP (ng/L) | NT-proB(ng/L) | p |
| --- | --- | --- | --- | --- |
| Control | 216±16 | 53.4±6.7 | 179.0±18.5 | 2.0E-03 |
| PTE(24h) | 207±21 | 73.6±8.4 | 227.4±11.0 | 1.0E-02 |
| Percent increase(%) | / | 38.0±1.6 | 27.8±7.1 | / |
